# Cross-Cultural Participation in Food-Related Activities and Quality of Life among Children with Celiac Disease

**DOI:** 10.3390/children10081300

**Published:** 2023-07-28

**Authors:** Sonya Meyer, Chiara Monachesi, Mara Barchetti, Elena Lionetti, Carlo Catassi

**Affiliations:** 1 Department of Occupational Therapy, Ariel University, Ariel 40700, Israel; 2 Celiac Disease Research Laboratory, Polytechnic University of Marche, 60123 Ancona, Italy; c.monachesi@pm.univpm.it; 3 Division of Pediatrics, DISCO Department, Polytechnic University of Marche, 60123 Ancona, Italy; mara.barchetti@gmail.com (M.B.); m.e.lionetti@univpm.it (E.L.)

**Keywords:** celiac disease, cross-culture, food-related activities, quality of life, questionnaire

## Abstract

Children with celiac disease may face challenges in managing a gluten-free diet during their daily interactions and activities. The objective of this study was to compare how children with celiac disease manage their gluten-free diet and participate in food-related activities in Italy and Israel and to assess their quality of life. The previously validated Children’s Activities Report (CD-Chart) and the Disease-specific Health-Related Quality of Life Questionnaire for Children with Celiac Disease (CDDUX) were administered in Italy to children aged 8–16 diagnosed with CD (*n* = 39). The results were compared to data that had been previously gathered from Israeli children with CD (*n* = 106). The CD-Chart demonstrated satisfactory internal reliability within each cultural group (Italy: α = 0.82; Israel: α = 0.76). Mann–Whitney U-tests indicated significant differences between the two groups. The Italian children exhibited a significantly higher preference for participating in the activities compared to the Israelis (*U* = 3283.50, *p* < 0.001). Nonetheless, the Italian children displayed a notable decrease in their level of involvement in the preparation required before engaging in different activities (*U* = 760.50, *p* < 0.001). Moreover, they exhibited significantly lower self-determination in this preparatory process compared to the Israeli children (*U* = 726.00, *p* < 0.001). Significant group differences were found between the CDDUX children’s self-reports and parents’ proxy reports in the Israeli group but not in the Italian group. The CD-Chart revealed both shared and distinct participation characteristics in daily food-related activities across different cultural contexts. By incorporating the CD-Chart and the CDDUX, healthcare professionals can emphasize crucial aspects of day-to-day health management and guide them in establishing suitable intervention objectives to enhance effective health self-management.

## 1. Introduction

Celiac disease (CD) is a common autoimmune disorder characterized by chronic inflammation of the small bowel. The condition is triggered by a permanent intolerance to gluten and primarily affects individuals with a genetic predisposition [1,2]. Gluten is a protein present in various grains (wheat, rye, and barley). In fact, the daily intake of gluten in the Mediterranean diet is about 10–20 g/day. Gluten’s main role is not primarily nutritional, as it lacks essential amino acids. However, it is critical in enhancing the cohesion of food items like bread and pasta [3,4,5].

Celiac disease (CD) is a chronic health condition and a persistent global medical issue, affecting an increasing number of individuals across their lifespans [6]. Until about 50 years ago, CD was regarded as an uncommon and typical childhood disease. Today, CD is widespread, with an estimated prevalence of approximately 0.6 to 1% in most populations. Over recent decades, the incidence of CD diagnoses has increased. This rise has been attributed to a combination of factors, including heightened awareness and testing and an increase in autoimmunity. There is regional variation in the different continents worldwide [3,7,8,9]. Certain countries, such as Saharan Africa and Japan, demonstrate a low prevalence of CD-predisposing genes and minimal gluten consumption [3]. Significant regional variations exist in Europe, with prevalence rates ranging from 0.3% in Germany to 2.4% in Finland, and the reasons for these differences remain unclear. Additionally, celiac disease is prevalent in developing countries, especially in North Africa and the Middle East. Throughout time, there have been noteworthy transformations in CD diagnosis, pathogenesis, and natural progression, leading to a continuous rise in the number of identified cases [10]. For example, prevalence in the United States has shown a five-fold increase in the frequency of CD from the 1960s to 2000 [11]. The prevalence of CD is considerably higher among females than in males (0.6% versus 0.4%) and is also higher among children compared to adults (0.9% versus 0.5%) [12]. The initial occurrence may occur shortly after transitioning from breastfeeding to solid foods, specifically during the introduction of gluten within the first two years of a child’s life. Nevertheless, celiac disease can manifest at any age, from the early stages of childhood, and persist throughout the lifespan. Diagnosing CD at any age can be difficult as symptoms exhibit considerable variation among individual patients [13]. CD is diagnosed by combining serology and histology, with or without duodenal biopsy [14,15,16].

Over the past decade, significant efforts have been dedicated to the pursuit of pharmacological treatments for CD [17]. Nevertheless, a lifelong gluten-free diet (GFD), eliminating all products containing wheat, rye, and barley, remains the only effective treatment for CD. The rigid and limiting GFD is complex and challenging to follow consistently and can negatively impact the quality of and affect social functioning, and adherence is estimated at 45–80% [1,2,18,19]. Ensuring the regular monitoring of individuals with CD who follow a GFD is crucial for evaluating their response to the diet, identifying any potential complications of CD, detecting associated autoimmune diseases, and pinpointing metabolic changes caused by the GFD [16]. For children with CD, adhering to a strict diet poses distinctive challenges in their daily activities and participation [20]. Food holds a central position in the daily routines of individuals of all age groups and cultures, and choices related to feeding and eating are influenced, among various factors, by medical considerations [21].

Active involvement in meaningful occupations in everyday life contributes to promoting, facilitating, supporting, and sustaining both health and quality of life [22,23]. The children’s activities report (CD-Chart) was developed in Israel to examine participation in food-related activities. The CD-Chart is a practical self-reported measure for collecting information from children about managing their health condition when participating in food-related activities, identifying their strengths and challenges, and promoting self-management, diet adherence, and well-being [20].

Research about health-related quality of life (HRQOL) among children with CD has been increasingly conducted in different countries over the past two decades (e.g., [24,25,26,27,28,29,30,31,32,33]. Recent European guidelines have recommended assessing the quality of life using validated disease-specific measures as part of the follow-up protocol for managing and following children with CD [34]. Children’s self-reported HRQOL using CD-specific HRQOL questionnaires have shown perceptions of poor to neutral HRQOL, while parents tend to underestimate the HRQOL of their children [2].

Cultural interpretations of health, religion, holidays, and comfort food can influence feeding and eating choices [21]. Culture is composed of a complex system of multiple universal constituents, such as language, values, traditions, and behaviors, that merge to create a whole. Culture influences all facets of everyday life, including habits and routines [35]. Among other things, culture shapes people’s daily activities and how they occupy their time, care for themselves, and enjoy life [36]. Therefore, the language and culture validation of research tools is necessary [37]. Due to variations in cultural values, individuals from different cultures often assign varying degrees of importance to different aspects of their lives. Consequently, validating research tools in terms of language and culture becomes essential [38]. Hence, this study aimed to (1) translate, culturally adapt, validate, and examine the generalizability and validity of the Celiac Disease-Children’s Activities Report (CD-Chart) [20] and (2) compare the participation in food-related activities characteristics and HRQOL of children with CD in Italy and Israel.

## 2. Materials and Methods

### 2.1. Participants and Procedure

**Figure 1:**
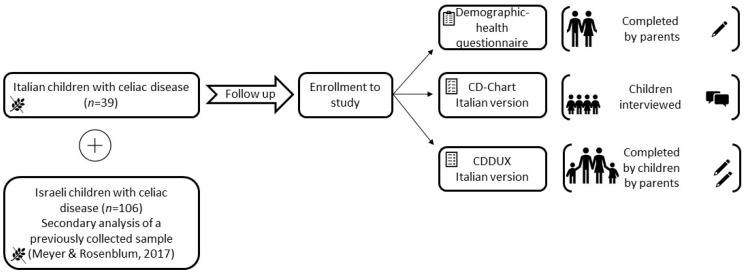
Methodology flowchart [20].

This prospective observational study included a sample of children with CD in Italy aged 8 to 16 years (*n* = 39). The Italian sample was compared to a previously collected larger sample of children with CD in Israel [20,32]. The original Israeli sample included children aged 8 to 18 years. To compare the samples, only children up to the age of 16 years were included in this secondary data analysis (*n* = 106) (Figure 1). The Italian sample consisted of children who were diagnosed with CD based on the ESPGHAN criteria. [39]. Participants in Italy were enrolled between January 2019 and February 2020 during a follow-up appointment at the Division of Pediatrics and Center for Celiac Research, DISCO Department, Marche Polytechnic University, Ancona. Inclusion criteria were children aged 8–16 years diagnosed with CD at least six months before enrollment in the study. Children with severe neurological disabilities were excluded. Italian children’s parents signed a written informed consent form before participation in the study. The study protocol was prepared by good clinical practice standards and approved by the Regional Ethics Committee (Approval number 2018 302/5920, 22 November 2018).

### 2.2. Measures

#### 2.2.1. Demographic Questionnaire

Parents in Italy completed a demographic-health questionnaire while waiting for the follow-up visit.

#### 2.2.2. Celiac Disease-Children’s Activities Report (CD-Chart)

The Celiac Disease-Children’s Activities Report (CD-Chart) [20] is a questionnaire for acquiring information about engagement in food-related activities among children with CD ages 8 to 18 years. The CD-Chart (available from https://osf.io/u3jg5 (accessed on 25 July 2023) comprises nine food-related activities that take place in three different surroundings: (1) Social environment (e.g., eating out with friends), (2) Close family environment (e.g., preparing a light meal/snack at home), and (3) Trip environment (e.g., participating in meals on overnight school excursions). Each activity is assessed across six dimensions: (a) activity (whether the child engages in the activity or not), (b) frequency (how often), (c) preference/liking (how much they like the activity), (d) preparation (whether participation in the activity requires special advance preparation), (e) involvement (the level of the child’s involvement in the preparation process), and (f) self-determination (the degree of importance the child places on performing tasks independently). Higher scores on the questionnaire indicate a greater frequency of activities, higher preference, increased involvement, and a stronger desire for independence. The CD-Chart was initially developed in English and Hebrew for administration in Israel. After translating the questionnaire into Italian, it was administered in Italy through an interview process as part of the follow-up appointment. Subtle cultural adaptation was made to one of the nine items; “Eating snacks handed out by the teachers at school (e.g., report card day)” was replaced by “Eating snacks brought to school to celebrate a friend’s birthday”. This adaptation was made, as it is not customary for teachers in Italy to hand out snacks. This adaptation did not change the affiliation of the item to the “Social environment”.

#### 2.2.3. Disease-Specific Health-Related Quality of Life Questionnaire for Children with Celiac Disease (CDDUX)

The 12-item Disease-specific Health-related Quality of Life Questionnaire for Children with CD (CDDUX) is a specific questionnaire for children with CD aged between 8 and 18 years for evaluating health-related quality of life (HRQOL) [24]. The CDDUX includes a self-report version for children to complete about how they perceive their HRQOL, and a parent-report version, for parents to prevail their perspective about how they think their child feels. The CDDUX collects HRQOL information organized in three subscales: (1) Diet (i.e., how the child feels about adhering to the dietary restrictions), (2) Having CD (i.e., how the child feels when offered food containing gluten or when thinking about food containing gluten), and (3) Communication (i.e., how the child feels when talking about CD). Responses are recorded on a five-facial expression scale interpreted as very bad, bad, neutral, good, and very good. The sum of the responses provides a final score, on a scale of 1 to 100, with higher scores indicating a better quality of life. The Italian version of the CDDUX was administered in Italy [31].

### 2.3. Data Analysis

The data were subjected to analysis using IBM SPSS Statistics version 29 (IBM Corp, Armonk, NY, USA). Descriptive statistics (means, standard deviations, and medians) were used to describe the participants’ demographic details. The internal reliability of the CD-Chart and CDDUX translated versions was determined with Cronbach’s coefficient alpha. The normality of distribution was examined using the Kolmogorov–Smirnov test. Due to not normally distributed data, non-parametric Mann–Whitney test was used to test for group differences in the CD-Chart and the CDDUX scale scores. The nonparametric Wilcoxon signed-rank test was used to analyze within-case differences between the CDDUX self and parent reports and the CDDUX’s twelve items. The significance level was set at 0.05.

## 3. Results

### 3.1. Demographics

The two cultural groups, from Italy and Israel, included eighty children (64.8% girls) aged 8.00–15.9 years (*M*_age_ = 11.56, *SD* = 2.27). No significant age difference was found between the two groups (*U* = 2096.50, *Mdn* = 11.33, *p* = 0.895) diagnosed with CD between six months to three years before recruitment participated in this study (*M* = 2.51. *SD* = 0.65). 

Parents reported CD diagnosis by means of laboratory tests and biopsy (77.2%) or only laboratory tests (22.8%), according to the ESPGHAN diagnostic guidelines [35]. The children in the Italian sample were diagnosed between the ages of 2–13 years, most between the ages of 6 and 11 years (53.8%). There was no significant difference between the two groups in the age of diagnosis (*U* = 2364.0, *Mdn* = 7.00, *p* = 0.183).

A comparison of demographic variables between the two groups is presented in Table 1.

### 3.2. The CD-Chart

#### 3.2.1. Internal Reliability

The CD-Chart’s internal reliability was determined by calculating Cronbach’s coefficient α (α = 0.76) for all nine items across the preference of participating in the activities domain in the entire sample (*N* = 145). Internal reliability was calculated in each cultural group (Italy: α = 0.82; Israel: α = 0.76).

#### 3.2.2. Differences in CD-Chart Performance between Cultural Groups

A Kolmogorov–Smirnov test of normality was conducted to determine whether the data were normally distributed. The results indicated that the data are not normally distributed. Therefore, a non-parametric Mann–Whitney test was used to compare groups. Significant differences were found between girls and boys in how often they participate in the activities (*U* = 1745.50, *Mdn* = 1.67, *p* = 0.007) and how much they enjoy participating (*U* = 1821.00. *Mdn* = 7.88, *p* = 0.017). An examination of each country showed no gender difference among the Italian children, yet significant differences were found in that the Israeli girls participated in food-related activities more often than the boys (*U* = 901, *Mdn* = 1.7, *p* = 0.006) and enjoyed participating more than the boys (*U* = 1020.00, *Mdn* = 7.63, *p* = 0.05). The median scores of the six CD-Chart dimensions were calculated across all nine items. The results indicated cross-cultural differences in preference, involvement, and self-determination. While the Italian children exhibited a significantly higher preference for participating in activities compared to the Israeli children, they were noticeably less engaged in the preparatory aspects required before engaging in various activities. Additionally, the Italian children displayed significantly lower self-determination compared to their Israeli counterparts (Table 2).

A further cross-cultural comparison was calculated for the activities in the “Social environment”, “Close family environment”, and “Trip environment”.

1. Social environment

The analysis of each dimension within the social environment factor revealed that Italian children expressed a significantly higher preference for participation in these activities compared to Israeli children. However, the Israeli children participated in a significantly greater number of activities more frequently, demonstrated increased involvement in the preparation process, and exhibited significantly higher levels of self-determination compared to the Italian children (Table 3).

2. Close family and Trip environments

As shown in Table 3, the Italian children demonstrated higher participation rates in close family food-related activities, and they derived more enjoyment from these activities compared to the Israeli children. Conversely, when it came to trip activities, the Italian children participated significantly more (*M* = 0.78) than their Israeli counterparts (*M* = 0.49), and they also exhibited a greater liking for these activities. However, the Israeli children displayed greater involvement in the preparation process and significantly higher self-determination levels in comparison to the Italian children (Table 3).

### 3.3. CDDUX

#### 3.3.1. Internal Reliability

The internal reliability of the CDDUX for the entire sample (*N* = 145) was deemed acceptable for both the child form (Cronbach’s coefficient α = 0.90) and the parent form (Cronbach’s coefficient α = 0.89). Table 4 presents the internal reliability of the translated versions of the CDDUX in Italian and Hebrew for each cultural group.

#### 3.3.2. Differences in CDDUX Scores between Cultural Groups

First, due to the broad age range of the children, correlations between HRQOL variables and age were tested, and no significance was found. In addition, no significant correlations were found between HRQOL variables and gender. The mean scores of the three CDDUX scales and the total scores were calculated for the children’s self-reports and parents’ reports between the two cultural groups (Table 5). 

To assess the normality of the data, a Kolmogorov–Smirnov test was conducted, indicating that the data was not normally distributed. Consequently, non-parametric Mann–Whitney tests were employed to compare the groups. The results revealed no significant differences between the cultural groups concerning the children’s self-reports in the diet (*U* = 2174.50, *Mdn* = 56.67, *p* = 0.631), having CD (*U* = 2369.50, *Mdn* = 46.67, *p* = 0.172), and communication scales (*U* = 1944.50, *Mdn* = 66.67, *p* = 0.581). Similarly, no significant differences were found in the parents’ proxy reports in the diet (*U* = 2311.00, *Mdn* = 50.00, *p* = 0.177) and communication scales (*U* = 1610.00, *Mdn* = 66.67, *p* = 0.063). A significant difference was found in the having CD scale (*U* = 2535.50, *Mdn* =46.67, *p* = 0.017), with better HRQOL reported on this scale by the Italian parents (*Mdn* = 53.33) than the Israeli parents (*Mdn* = 46.67). 

#### 3.3.3. Comparison between Children’s and Parents’ CDDUX Reports in Italy and Israel

A Wilcoxon signed-rank test to calculate within-subject (child-parent) differences showed significant differences in the total CDDUX scores between the children’s self-reports and the parents’ proxy reports about their children in the entire sample (*Z* = −2.891, *p* = 0.004). No significant differences were found when separately examining the Italian sample (*Z* = −0.893, *p* = 0.372). An examination of the Israeli sample showed a significant difference in the total score between children’s and parents’ reports (*Z* = −2.88, *p* = 0.004). Among the Israeli participants, the children rated their HRQOL significantly higher than their parents’ rating on the diet scale (Children: *M* = 54.28, *Mdn* =55.00, *SD* = 18.02; Parents: *M* = 49.43, *Mdn* = 48.33, *SD* = 14.95, *Z* = −3.14, *p* = 0.002), and the having CD scale (Children: *M* = 48.55, *Mdn* =46.67, *SD* = 15.08; Parents: *M* = 44.67, *Mdn* = 46.67, *SD* = 12.95, *Z* = −2.33, *p* = 0.020). No significant difference was found in the communication scale (Children: *M* = 70.44, *Mdn* = 73.33, *SD* = 20.47; Parents: *M* = 69.94, *Mdn* = 73.33, *SD* = 16.92, *Z* = −0.56, *p* = 0.575).

## 4. Discussion

The main objective of this study was twofold: firstly, to translate and culturally adapt the CD-Chart into Italian, assessing its cultural relevance and comparing the outcomes with those previously obtained in Israel, and secondly, to compare the health-related quality of life (HRQOL) among children with CD in Italy and Israel, as reported both by the children themselves and their parents.

Our results confirm the good internal reliability of both questionnaires in the two groups of children from different cultural backgrounds, indicating that these questionnaires can be used in different cultural contexts. However, the results of this study reveal numerous differences in how children participate in food-related activities in the two countries. Italian children demonstrate a greater enjoyment of social food-related activities compared to their Israeli counterparts. On the other hand, while the Israeli children have a lower level of enjoyment in these activities than the Italian children, they participate more frequently, actively involve themselves in the preparation process beforehand, and express a stronger desire for independence in the preparation. Interestingly, social participation is stressed as an essential indicator of health and well-being [40]. Further, striving for independence is a crucial developmental stage that occurs from childhood through adolescence that is an important required skill for managing the GFD [41,42]. The active involvement of children in activities and assuming responsibilities contributes to the support and maintenance of their well-being [22]. Although the Italian children participated more often in trip activities than the Israeli children, the Israeli children showed similar tendencies for more involvement and more of a desire for independence in these activities, as found in the social activities. The different cultural environments, social demands, interpretations of health, and daily habits and routines may be the sources of this significant difference [21,35]. Nevertheless, these differences were absent in the close family environment. This may reflect the similarities in family habits, routines, and traditions between the two cultures. Cultural validation and semantic understanding are essential because culture comprises an intricate network of universal elements, including language, values, traditions, and behaviors, which merge to form a cohesive entity. Different cultures tend to place varying degrees of significance on various aspects of life, highlighting the need for validation and understanding [37]. 

Both cultural groups showed medium to high HRQOL scores. This reflects that in both cultures, the children perceive their health-related quality of life to be relatively good despite the challenges of facing numerous demanding food-related situations in everyday life [20,24,43]. A significant difference was found between the two groups in the parent’s report on the Having CD scale, with a higher rating among the Italian parents than the Israeli parents. Still, both groups reported a medium level of HRQOL. Differences between the two cultures occurred when comparing child-parent HRQOL reports. Surprisingly, no differences were found among the Italian child-parent perceptions of HRQOL. Differences were apparent among the Israeli children and parents. This finding aligns with previous studies comparing self and parent reports. Often, differences are found between child and proxy HRQOL reports. Parents habitually report lower HRQOL than children’s self-report, and health professionals may report higher HRQOL [25,44,45,46,47]. The Israeli children rated their HRQOL significantly higher than their parents reported on the Diet and Having CD scales, while no differences were found on the communication scale. Although CD is an externally “invisible” disease, its daily management requires cooperation and communication with the surrounding environment [48]. The similarity in the children’s and parents’ perception of the need to communicate with others about CD may reflect the understanding of the importance of advocating and communicating the health condition to the children’s various environments. The Italian parents’ perception of their children’s HRQOL is possibly related to global variability in CD awareness, including in Italy and Israel [12,49]. 

## 5. Limitations and Future Research

The study’s main limitation is the small sample size, which hindered a more precise differentiation between younger children and older adolescents. Data collection in Italy began before the COVID-19 pandemic; however, the outbreak significantly affected enrollment, resulting in substantial limitations. Consequently, we had to rely on the available sample for our study. Nonetheless, it has been suggested that a sample size of 15 to 30 can still provide preliminary evidence for internal consistency in health status questionnaires [50]. Moreover, the Pediatric Center in Italy typically handles children up to the age of 16, unlike the original larger Israeli sample, which included children up to 18. Additionally, the data in Israel was collected in 2015, a few years before the Italian data collection. As awareness of CD continues to grow over time, the time span between data collection periods may have influenced the results and perceptions of the disease. To further our understanding of the cultural factors influencing the HRQOL of celiac patients, future studies could incorporate larger samples and diverse cultural groups. Furthermore, it may be beneficial to assess the CD-Chart, with appropriate adaptations, for other food-related chronic diseases that necessitate self-management strategies. 

## 6. Conclusions

In summary, the CD-Chart showed reliability in Italy and Israel in both languages. The CD-Chart showcased both shared and distinct participation characteristics in diverse contexts of daily food-related activities across different cultures. By illuminating essential aspects of day-to-day health management, the CD-Chart can aid clinicians in establishing appropriate intervention goals to enhance effective health self-management. Our study supports the CD-Chart and the CDDUX validation in managing CD. Both questionnaires can be valuable tools for healthcare professionals involved in CD follow-up. Integrating these tools in follow-up can promote a better understanding of each child’s or adolescent’s specific challenges, direct them to suitable supports, and promote their well-being and HRQOL.

## Figures and Tables

**Table 1:** Comparison of demographic variables.

Variable	Italy *n* = 39	Israel *n* = 106	χ^2^	*p*-Value
Gender (girls)	28 (71.8%)	66 (62.3%)	1.14	0.287
Living				
City	31 (79.5%)	71 (67.0%)	2.14	0.144
Community/countryside	8 (20.0%)	35 (33.0%)		
Child’s general health				
Good	39 (100.0%)	102 (96.2%)	1.51	0.219
Reasonable	0	4 (3.8%)		
Type of Diagnosis				
Laboratory tests and biopsy	16 (41.0%)	96 (90.6%)	39.80	<0.001 ***
Laboratory tests only	23 (59.0%)	10 (9.4%)		
Time since diagnosis				
6–12 months	9 (23.1%)	3 (2.8%)	18.63	<0.001 ***
1–3 years	15 (38.5%)	32 (30.2%)		
Over three years	15 (38.5%)	71 (67.0%)		
Place of diagnosis				
Hospital	39 (100%)	80 (75.5%)	11.66	<0.001 ***
Community doctor	0.0	26 (24.5%)		
Present CD medical status				
Negative antibodies	25 (64.1%)	77 (72.6%)	5.30	0.151
Antibodies in decline	11 (28.2%)	20 (18.9%)		
Positive antibodies	3 (7.7%)	3 (2.8%)		
Level of adherence				
High	37 (94.9%)	103 (97.2%)	0.452	0.501
Good	2 (5.1%)	3 (2.8%)		
Frequency of follow-up				
Once every six months	19 (48.7%)	25 (23.6%)	3.67	0.299
Once a year	17 (43.6%)	55 (51.9%)		
Once every few years	3 (7.7%)	21 (19.8%)		
No follow-up	0.0	5 (4.7%)		
Additional health issues				
No	30 (76.9%)	84 (79.2%)	0.091	0.762
Yes	9 (23.1%)	22 (20.8%)		
Regular use of medication				
No	34 (85.0%)	35 (87.5%)	0.105	0.745
Yes	6 (15.0%)	5 (12.5%)		
Mother’s health status				
Good	33 (84.6%)	102 (96.2%)	5.99	0.014 *
Reasonable	6 (15.04%)	4 (3.8%)		
Bad	0.0	0.0		
Father’s health status				
Good	33 (84.6%)	96 (92.3%)	1.960	0.375
Reasonable	5 (12.8%)	7 (6.7%)		
Bad	1 (2.6%)	1 (1.0%)		
Parents personal status				
Married	34 (85.0%)	97 (91.5%)	6.89	0.142
Separated	2 (5.1%)	0.0		
Divorced	2 (5.1%)	3 (2.8%)		
Other	2 (5.1%)	6 (5.7%)		

Note. CD = celiac disease, χ^2^ = Cross Tabulations with a Chi-Square Test of Independence, * *p* < 0.05, *** *p* ≤ 0.001.

**Table 2:** Median, Range, and Mann–Whitney Test Results for CD-Chart Dimensions’ Group Differences.

	Italy (*n* = 39)		Israel (*n* = 106)			
CD-Chart Dimensions	*Mdn*	Min–Max	*Mdn*	Min–Max	Mann-Whitney *U*	*p*-Value
Activity (scale = 0–1)	8.00	5.00–9.00	8.00	5.00–9.00	2002.00	0.761
Frequency (scale = 1–4)	1.63	1.14–2.00	1.71	1.25–2.44	1683.50	0.087
Preference (scale = 1–10)	8.88	5.86–10.00	7.63	3.88–9.75	3283.50	<0.001 ***
Preparation (scale = 0–1)	1.00	1.00–1.00	1.00	0.83–1.00	2164.50	0.169
Involvement (scale = 1–5)	2.25	1.00–4.33	3.29	1.71–4.60	760.00	<0.001 ***
Self-determination (scale= 1–3)	1.63	1.00–2.22	1.71	1.00–2.71	726.00	<0.001 ***

Note. CD-Chart = Celiac Disease-Children’s activities report, *Mdn* = median, *** *p* < 0.001.

**Table 3:** Median, Range, and Mann–Whitney Test Results for CD-Chart Factor/Component Group Differences.

	Italy (*n* = 39)		Israel (*n* = 106)		Mann-Whitney *U*	*p*-Value
Factor/Component and Dimension	*Mdn*	Min–Max	*Mdn*	Min–Max		
Social environment						
Activity	1.00	0.40–1.00	1.00	0.60–1.00	1485.00	≤0.001 ***
Frequency	1.50	1.00–2.33	1.80	1.00–2.80	955.50	<0.001 ***
Preference	9.00	5.20–10.00	7.60	3.20–10.00	2977.50	<0.001 ***
Preparation	1.00	1.00–1.00	1.00	0.75–1.00	2164.50	0.169
Involvement	2.00	1.00–4.25	3.40	1.50–4.75	617.00	<0.001 ***
Self-determination	1.50	1.00–2.25	2.00	1.00–3.00	620.00	<0.001 ***
Close family environment						
Activity	1.00	0.50–1.00	1.00	0.50–1.00	9092.00	0.724
Frequency	2.50	1.00–3.50	2.00	1.00–4.00	3158.50	<0.001 ***
Preference	9.00	6.50–10.00	8.50	4.00–10.00	2587.50	0.019 *
Preparation	1.00	1.00–1.00	1.00	1.00–1.00	2067.00	1.00
Involvement	3.00	1.00–4.50	2.00	1.00–5.00	2223.50	0.419
Self-determination	2.00	1.00–2.50	2.00	1.00–3.00	1733.50	0.100
Trip environment						
Activity	0.50	0.00–1.00	0.50	0.00–1.00	2595.00	0.012 *
Frequency	1.00	1.00–1.00	1.00	1.00–3.00	1139.00	0.087
Preference	9.00	5.50–10.00	6.00	1.00–10.00	2137.00	<0.001 ***
Preparation	1.00	1.00–1.00	1.00	1.00–1.00	1224.00	1.00
Involvement	2.00	1.00–4.00	4.00	1.00–4.50	646.50	<0.001 ***
Self-determination	1.50	0.50–2.00	2.00	1.00–3.00	789.50	<0.001 ***

Note. CD-Chart = Celiac Disease-Children’s activities report; * p < 0.05; *** *p* ≤ 0.001.

**Table 4:** Internal Reliability of the CDDUX.

	Child Form (*n* = 145)		Parent Form (*n* = 145)	
	Italy α=	Israel α=	Italy α=	Israel α=
CDDUX Diet (6 items)	0.82	0.9	0.91	0.89
CDDUX Having CD (3 items)	0.7	0.68	0.61	0.6
CDDUX Communication (3 items)	0.86	0.92	0.83	0.92
CDDUX Total (12 items)	0.86	0.91	0.91	0.89

Note. CDDUX = Disease-specific Health-Related Quality of Life Questionnaire for Children with Celiac Disease, Cronbach’s alpha = α.

**Table 5:** CDDUX Scores in Italy and Israel.

	Children (*n* = 145) *M* (*SD*)		Parents (*n* = 145) *M* (*SD*)	
CDDUX Variables	Italy (*n* = 39)	Israel (*n* = 106)	Italy (*n* = 39)	Israel (*n* = 106)
Diet	55.47 (16.39)	54.28 (18.01)	53.51 (15.60)	49.43 (14.95)
Having CD	52.65 (14.25)	48.55 (15.08)	52.46 (14.88)	44.97 (12.95)
Communication	69.40 (17.95)	70.44 (20.47)	64.56 (14.47)	69.94 (16.92)
Total score	58.24 (13.00)	56.89 (15.31)	56.01 (13.09)	53.44 (12.26)

Note. 1–20 = very bad, 21–40 = bad, 41–60 = neutral, 61–80 = good, 81–100 = very good; M = mean; SD = standard deviation.

## Data Availability

The data presented in this study are available on request from the corresponding author. The data are not publicly available due to ethical restrictions.
